# SARS-COV-2 as an artificial creation: scientific arguments and counterarguments

**DOI:** 10.25122/jml-2020-0175

**Published:** 2021

**Authors:** Shermaine Yee, Ching Siang Tan, Abdullah Khan, Kah Seng Lee, Bey Hing Goh, Long Chiau Ming

**Affiliations:** 1. Faculty of Medicine, Quest International University Perak, Ipoh, Perak, Malaysia; 2. School of Pharmacy, KPJ Healthcare University College, Nilai, Negeri Sembilan, Malaysia; 3. Faculty of Pharmacy, University of Cyberjaya, Cyberjaya, Selangor, Malaysia; 4. Biofunctional Molecule Exploratory Research Group (BMEX), School of Pharmacy, Monash University Malaysia, Bandar Sunway, Malaysia; 5. Pengiran Anak Puteri Rashidah Sa’adatul Bolkiah Institute of Health Sciences, Universiti Brunei Darussalam, Gadong, Brunei Darussalam

## Introduction

Discussions and questions on the origin of the virus that causes coronavirus disease 2019 (COVID-19) have been frequently raised and widely debated among all communities since the first large-scale outbreak was detected in December 2019. COVID-19 has rapidly acquired a pandemic status, leading to the death of more than six hundred thousand people all across the world at the time of writing. Thus, it is crucial to discover its origin to prevent another viral outbreak [1]. The main theories regarding the origin of severe acute respiratory syndrome coronavirus 2 (SARS-COV-2) are that it arose naturally, as a zoonotic infection, that it was deliberately engineered as a bioweapon [2, 3], or that it has accidentally leaked out from a bio lab in China by researchers that were studying a virus related to the SARS coronavirus. Therefore, this leads to a highly debatable question: is COVID-19 a man-made or a naturally occurring disease?

Coronavirus is an enveloped virus with a single-stranded, positive-sense ribonucleic acid genome. It contains a total of four genera: alpha, beta, gamma, and delta. The beta genus is classified into four lineages from A to D. Scientific evidence showed that the virus causing COVID-19 belongs to the B lineage of the beta coronavirus group, which is the same group of another epidemic-causing virus, severe acute respiratory syndrome coronavirus (SARS coronavirus). Because of this similarity, it was named SARS-COV-2 [4, 5].

### Argument: a naturally mutated virus 

Considering the previous pandemic, it can be inferred that COVID-19 is most probably a disease due to zoonotic transmission. This is supported by studies showing that SARS coronavirus originates from direct contact between humans and civets. Another novel coronavirus, Middle East respiratory syndrome coronavirus (MERS-CoV), has also been discovered because of its direct transmission to humans from dromedary camels [6]. Retrospective analysis of a few early confirmed cases in Wuhan last year shows that this contagious disease is transmitted through direct person-to-person contact. According to the scientific evidence available, bats are inferred to be the host animals responsible for starting the transmission chain of this virus. Up to 96% similarity was found between SARS-COV-2 and the bat coronavirus using whole-genome analysis [7]. This theory was supported by other studies that have been summarized in detail elsewhere [8, 9]. A study supported this suggestive finding by using sequence-based analysis and artificial intelligence on the genomic sequence of the novel coronavirus [10]. Snakes were found to be the most probable wildlife repository for the virus compared with other animals [11]. Conversely, another study suggested that SARS-COV-2 could have originated from bats but with pangolins as the possible intermediate hosts [12]. 

In addition to the theory that COVID-19 is transmitted from bats to humans, it was found that natural selection is the reason behind the binding of the mutated SARS-COV-2 spike protein to the human-like angiotensin-converting enzyme 2 (ACE2) receptor. Besides that, the polybasic cleavage site of this novel coronavirus shows a possibility that it arises through a natural-occurring evolutionary process. The criterion for the enhancement of binding between a precursor virus and the ACE2 receptor is concluded elsewhere [13]. On the other hand, there was an inference on the origin of SARS-COV-2 where the precursor of the virus acquired genomic features through adaptation after a zoonotic transmission and the insertion of its polybasic cleavage site during direct transmission between humans [14].

### Counterargument: a man-made virus

While study results proposed and supported the theory of COVID-19 as a resultant of a naturally-occurring event, there is another suggestive theory stating SARS-COV-2 is an intentionally engineered virus that was unintentionally released from a bio lab in Wuhan, China. The origins of the epidemic and the “probable” bat source have raised skepticism. The Wuhan Institute of Virology report stated that the source – bat-related coronavirus – was found in the southern area of Yunnan, same as the origin of the SARS-COV-2 outbreak [11]. Comparatively, the adjacent Wuhan Institute of Virology had raised a higher possibility of this disaster pandemic by spending $44 million on the National Biosafety Laboratory (Level 4). Naturally, the origins of the epidemic were investigated. All the studies of Shi Zhengli, the lead virologist from the institute, on bat related coronaviruses were centered in the southern, subtropical areas of Yunnan. However, the outbreak occurred in Wuhan, which is almost 900 km from Yunnan. This cliffhanger seems to point to a possibility that this novel strain of coronavirus was leaked out artificially. Even though the Chinese government discredited the possibility of a lab origin based on genetic studies, the distance of the epicenter from the bat caves raises questions. 

Moreover, it is widely known that studies involving the study of the transmission of bat SARS-like coronaviruses using cell culture and animal models such as laboratory mice have been conducted for many years all across the world. Based on the scientific evidence, it is possible that an inadvertent laboratory released the progenitor of SARS-COV-2. Theoretically, mutation of the receptor-binding-domain (RBD) of SARS-COV-2 through adaptation during its passage in cell culture is possible since this was observed in previous SARS coronavirus passage studies [15]. However, a recent study provided a more parsimonious explanation on the alterations of the SARS-COV-2 receptor-binding domain (RBD) through recombination or mutations with more robust evidence [16]. The mechanism of both the polybasic cleavage site and O-glycosylation also raised a disagreement against the theory of culture-based scenarios being the origin of COVID-19. There is no detailed explanation and scientific evidence on the hypothesis regarding the generation of SARS-COV-2 through cell culture or animal passage, which would have required prior isolation of a precursor virus with high similarity in the genomic sequence. The RBD of viruses from Rhinolophus affinis bat and Malayan pangolins (Manis javanica) have a remarkably lower affinity to the angiotensin converting enzyme 2 (ACE2) receptors, albeit the two sources contain coronaviruses similar to SARS-COV-2 [14]. Results from other non-coronavirus studies have demonstrated that the criterion for generating a polybasic cleavage site is either the repeated passage of the virus in cell culture or animals with ACE2 receptors similar to those of humans, which were also not discussed in detail in the context of SARS-COV-2.

Furthermore, another research suggested that involvement of the immune system is needed for the generation of O-glycosylation in the virus. Therefore, it is improbable for it to have happened as a result of cell-culture passage [17]. Although no scientist has come forth with any definite evidence that proves human manipulation of the virus using any genetic engineering method, there are situations involving human intervention that have been reported [18]. It must be noted that some important features of the spike protein on SARS-COV-2 have also raised suspicions on whether the virus is man-made [19, 20]. This is because both S1 and S2 sites of the spike protein demonstrated optimal portions, which facilitates the penetration of the virus’ RNA into the living cell that could weaken the host defense of the host [2]. Besides, similar sequences were also indicated in the proteins endonuclease (nsp15) and 2′ -O-MTase (nsp16), among other sites of SARS-COV-2 [21].

### Final perspectives

Many hypotheses on the original source of SARS-COV-2 and COVID-19 have been made, which still lack concrete evidence that supports their statement. Among them, the theory involving recombination, convergence, and adaptation of SARS-COV-2 have been put in the limelight, suggesting a possibility in the evolutionary pathway for SARS-COV-2 [22]. Most scientific reports believe that the polybasic cleavage site and mutation of the spike proteins are the mechanisms behind the adaptation of this beta coronavirus group of SARS-COV-2 to humans [14]. The alterations in the receptor-binding domain of the surface protein (S) of SARS-COV-2 result in its effective binding to human ACE2 receptors, especially in the human respiratory airway, which increases the transmission ability of the virus. Moreover, results of a recent retrospective study conducted in Zhejiang, China, showed one amino acid position loss and four single amino acid mutations in SARS-COV-2 with greater similarity to humans than viruses [23]. Apart from that, genomic and strong evidence of the similarity between SARS-COV-2, bat-coronavirus, and pangolin-coronavirus at the whole genome level has also been discovered. Alterations of the S1-2 junction of coronavirus, including mutations, insertions and deletions, demonstrates that the generation of polybasic cleavage sites is achieved through a natural evolutionary process [14]. 

In conclusion, all these specific features observed in SARS-COV-2 helps scientists to rule out the idea that this pandemic caused by the novel coronavirus is the result of a man-made action that could be either engineered in the laboratory or further created as a bioweapon out of conspiracy. Recent discoveries revealed evidence of the presence of the virus around the world before it emerged in Asia. There is growing evidence of its true origin as a global organism that was waiting for favorable conditions to emerge instead of originating in China. Recent testing of sewage in Barcelona had suggested that the virus may have been present in the Spanish city in March 2019, many months before China identified the pathogen in the city of Wuhan in December 2019. Based on the results available, it is most probably that this is a natural-born virus that emerged from an animal host, most likely a bat, without any direct pieces of evidence about its intermediate host. Nevertheless, researchers are yet to find a definitive answer to which animal serves as an intermediate host for this virus and disease. Besides, questions on the role of SARS-CoV-2 in T cells have been raised, especially with evidence from recent postmortem findings of its preferential impact on CD 4+ and CD 8+ T cells [24–26]. Therefore, further studying of all microorganisms is mandatory in order to understand how they evolve, how they live, and how they transmit, which may be the key solution in hindering the spread of COVID-19.

## Acknowledgments

### Conflict of interest

The authors declare that there is no conflict of interest.
